# Performance Study of Lightweight Insulating Mortar Reinforced with Straw Fiber

**DOI:** 10.3390/ma16062266

**Published:** 2023-03-11

**Authors:** Xiao Zhang, Weitao Liu, Shuo Zhang, Jiaoyun Hou

**Affiliations:** 1 College of Safety and Environmental Engineering, Shandong University of Science and Technology, Qingdao 266590, China; zhangxiao19965718@163.com (X.Z.);; 2 State Key Laboratory of Mining Disaster Prevention and Control Co-Found by Shandong Province and the Ministry of Science and Technology, Shandong University of Science and Technology, Qingdao 266590, China; 3 The Fifth Exploration Team of Shandong Coalfield Geology Bureau, Jinan 250100, China

**Keywords:** straw fiber, compressive strength, SEM images, thermal insulation, numerical simulation

## Abstract

The current research aimed to develop lightweight, environmentally friendly mortar materials using crop straw fibers with better insulation properties. The lightweight mortar samples were tested for moisture content, thermal conductivity and compressive strength on days 3, 7 and 28, respectively. Scanning electron tomography (SEM) was performed on the fiber–matrix bonding interface and internal fiber structure. The permeability rating was also measured to check the impermeability of the lightweight fiber mortar. Due to the high hygroscopicity of plant fibers, the thermal conductivity of the mortar was high at the initial molding stage; the thermal conductivity measured at day 28 decreased with increasing fiber content, while the mechanical properties gradually decreased. The impermeability test showed that the straw fiber mortar had better impermeability than the standard mortar. However, with the addition of 2% of 10 mm long fibers, we increased the compressive strength and thermal insulation properties. Numerical simulations verified that the fiber insulation mortar has good thermal insulation properties in high-temperature tunnels.

## 1. Introduction

In recent years, increasing numbers of tunnels have been built at extremely high temperatures, the most representative of which are tunnels for resource exploitation at deeper levels. Excessive airflow temperatures in high-temperature tunnels seriously threaten tunnel excavation safety and workers’ health [1]. In high-temperature areas, mechanical refrigeration systems combined with underground zone cooling units reduce the airflow temperature [2]. This technology relies on the rational configuration and application of large refrigeration equipment and has high operating costs and low efficiency. The surrounding rock mass is the largest heat source in high-temperature tunnels, so wall insulation becomes the key to cooling. In this context, thermal insulation materials provide a new method of controlling tunnel thermal hazards.

Inorganic fiber concrete materials and their structural health monitoring have been developed well. D.G. Aggelis et al. [3] used acoustic emission techniques to detect and locate cracks, measure crack strength and characterize crack types, and the results were quite accurate. Maristella E. Voutetaki et al. [4] investigated the effect of adding synthetic fibers on concrete compression behavior and damage detection procedures and developed a reliable method for quantitative damage assessment. Manjunath V. Bhogone et al. [5] conducted fracture tests on concrete beams with a 0.66% volume fraction of discrete fiber reinforcement. The data showed that the synthetic fibers had high resistance to crack expansion at early stages; the residual tensile strength was significantly higher in the case of co-blended macro- and micropolypropylene fibers. Petr Lehner [6] found better shear performance for longer fibers when performing crack opening displacement tests on fibrous concrete specimens. Yewei Jiang [7] determined the optimal combination of insulation material doped with basalt fibers by orthogonal tests and data analysis, i.e., 45% doped basalt fibers with 6 mm basalt fiber length and 20% doped ceramic particles. The performance indexes of the best comprehensive combination of insulation materials doped with basalt fibers include a density of 1200 kg/m^3^, thermal conductivity of 0.151 W/(m·°C) and compressive strength of 9.7 MPa. Agathon [8] investigated the addition of glass wool fibers to asphalt concrete to enhance performance. Glass fibers, asphalt binder and aggregates were added to the mix for laboratory tests. The mixture with 0.3% total fiber content outperformed the other mixtures by increasing the indirect tensile strength, toughness, deformation strength and tensile strength ratios by 13%, 6%, 25% and 8%, respectively. Good progress has been made in applying fiber concrete materials for general building insulation. However, synthetic-based insulating fiber materials have many shortcomings and hazards. Glass fiber and asbestos fibers produce floating silica dust, glass wool dust and broken glass dust during production and on-site construction [9]. These dust specks endanger workers’ health, causing allergic skin diseases, respiratory diseases and even cancer [10]. Basalt fibers are widely used in defense security facilities, with small production volumes and high production costs [11]. They are an essential strategic material that is entering general civilian construction. The production of these synthetic-based insulating fiber materials is based on non-renewable resources. The production process produces a large amount of industrial waste and carbon dioxide, which is contrary to sustainable development. Today, the high cost of traditional building raw materials is also an important factor affecting the development of construction. The disadvantage of inorganic fibers is something we have to face. Following a sustainable development strategy, we seek a new fiber to replace synthetic fiber. Agricultural straw fibers have unique natural advantages in terms of comprehensive sources, abundant raw materials and low prices, and they also enhance the mechanical properties of composite materials [12,13,14]. Applying agricultural straw fibers for building wall insulation can reduce reliance on non-renewable materials, pollutant emissions, greenhouse gas emissions and improve biodegradable behavior [15].

Several studies have been published on plant fiber-based composites. Cavalcanti [16] used alkali-treated hemp fibers as natural fiber reinforcement to manufacture epoxy resin composites with a fiber volume fraction of 30%. The results showed that incorporating natural fibers in the composites significantly improved the mechanical properties of the materials. Muhammad [17] investigated the contribution of plant fibers (i.e., wheat straw) in improving the properties and capabilities of reinforced concrete. The addition of wheat straw to reinforced concrete increased flexural strength (up to 7.5%), energy absorption (up to 30.4%) and toughness index (up to 11.1%), as well as showing better crack arrest mechanisms. Tiezhi Zhang [18] found, using SEM micrographs, that jute fibers are able to hinder the propagation and connection of existing cracks as well as withstand some of the tensile stresses during loading, resulting in reinforcement, toughening and crack resistance. Straw bales have undeniable advantages in terms of low environmental loads, low energy consumption for production and favorable thermal engineering properties as part of the main load-bearing member or load-bearing structure [6]. Hanifi Binici [12] added different masses of sunflower and wheat straw to a mixture of gypsum and vermiculite to make compressed insulation. The proposed insulation has a thermal conductivity of 0.063–0.334 W/(m·°C), which has great potential for thermal insulation. Muhammad Asim [19] studied the effect of jute, coconut and sugarcane fibers with different fiber contents on the compressive strength and thermal conductivity of concrete and proposed a trade-off method for the thermal and mechanical parameters of natural-fiber-reinforced concrete. Fotini Kesikidou [20] studied the behavior of jute, coconut and kelp of the same length in the mortar; regardless of the type of fiber, the flexural strength and fracture energy of the mortar was significantly enhanced. The shape stability of the samples was preserved even after fracture and crack formation. Most scholars have studied the mechanical properties and thermal insulation properties of plant-fiber-reinforced composites with different contents added, but the added plant fibers have only one fixed length or even an average length, and few studies have considered both the effects of different fiber contents and lengths on the properties of the reinforced materials.

Kaiming [21] used numerical simulations to study the Sichuan–Tibet Railway tunnel’s high-temperature and humidity environment. Air temperature and relative humidity data were collected at the same locations in the tunnel exit cross-section, and the field measurements were found to agree with the simulated results. Yicai Liu [22], while studying the ventilation of an underground hydroelectric power plant, measured the air characteristics in different areas using tools such as a hygrometer, infrared thermometer and anemometer to check the accuracy of the simulation. The data from the air inlet agreed very well with the measured values and showed a similar trend. The data from the air outlet can greatly reflect the accuracy of the cooling effect of the simulated heat source and air conditions. COMSOL numerical simulations can reasonably implement temperature field simulations.

In China, straw stalks are produced in large quantities and are cheap to burn in the fields, which is very wasteful and pollutes the environment. This study aims to develop a lightweight straw fiber insulation mortar to be sprayed on the surface of high-temperature tunnels to achieve a cooling effect. Different mass proportions and lengths of straw fibers were used to make the lightweight insulation mortar. The experimental program focused on testing the performance of the samples on the third, seventh and twenty-eighth days of the hardening process, including moisture content, compressive strength, thermal conductivity and impermeability. Finally, the thermal insulation effect of the straw fiber mortar was verified using COMSOL (Stockholm, Sweden) numerical simulation software(COMSOL Multiphysics 5.6).

## 2. Materials and Experimental Testing

### 2.1. Materials

The constituent materials of the straw fiber lightweight mortar include cement, fly ash, straw fiber, natural river sand with a particle size of less than 1 mm, ceramic particles with a particle size of 2 to 3 mm and glass beads with a particle size of 0.5 to 1 mm. The cement we used was ordinary silicate cement. Plain silicate cement is a hydraulic cementitious material made of finely ground silicate cement clinker; the main components are CaO, SiO_2_ and Al_2_O_3_. Plain silicate cement has high strength, good frost resistance, dry reduction, better carbonation resistance and poor corrosion resistance. Fly ash is a fine ash recovered from the flue gas after coal combustion. The main components of fly ash are SiO_2_, Al_2_O_3_, FeO, Fe_2_O_3_, CaO, TiO_2_, etc. We used river sand with a fineness modulus of 2.78. The main component of river sand is SiO_2_. River sand has low mud content, rounded particles, smooth surface and good fluidity. Shale ceramic pellets are made of natural rock-shale as a raw material, refined by high temperature and roasting. They are light, compressive, heat preservers, shockproof, and non-radiative, among other advantages suitable for building energy-saving materials. Glass beads are made of selected mineral sand with special particle size, which is expanded under the heating of an electric furnace; they are a new type of environmentally friendly lightweight inorganic insulation material which are have excellent performance in heat preservation, heat insulation and fire prevention. The main chemical composition of glass beads includes SiO_2_, AI_2_O_3_ and CaO. Straw fiber was taken from rice straw stalks. The main components of straw fiber are cellulose, hemicellulose, lignin and wax.

### 2.2. Preparation of Straw Fiber

**Figure 1:**
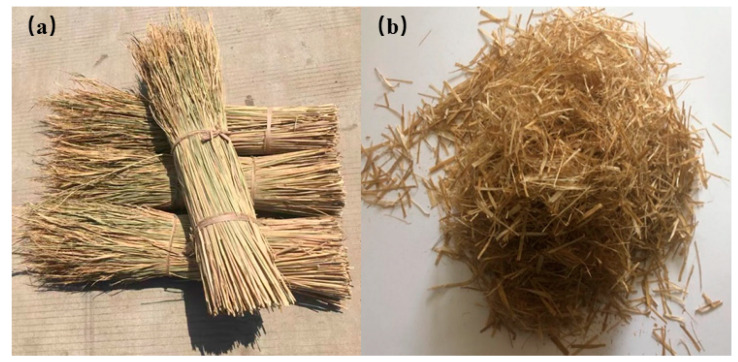
(**a**) Natural straw; (**b**) fibers.

The rice straw fibers were prepared from rice straw according to the suggested procedure [23]. The fiber was dried in an oven, and then the straw was treated with 4 g/L NaOH/H_2_O solution at 90~100 °C for 4~5 h, with continuous stirring (160/min). The straw fibers were washed with distilled water. Then, they were manually cut into small pieces of 5 mm, 10 mm, and 15 mm and crushed in a grinder to screen the regular fibers for use. The thickness and width of a straw fiber measured by a vernier caliper were about 0.5 mm and 1.5 mm, respectively. Figure 1 shows natural and prepared straw fiber.

### 2.3. Preparation of Straw Fiber Mortar Samples

**Figure 2:**
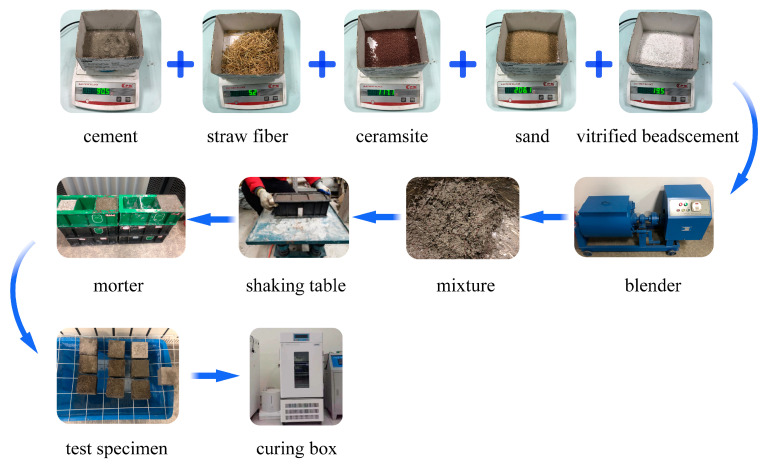
Flow chart of concrete sample preparation.

The ratio of the insulation mortar was derived after several tests and adjustments in combination with specifications and experience [24]. The mass ratio of fly ash, ordinary silicate cement, river sand and ceramic particles were fixed as 1:4:11.5:6.5. Cement and fly ash are binder materials, and the water to binder ratio is 0.55. Dried glass beads accounted for 50% of the volume of the mixture. The fibers were added to the mixture in different proportions (2%, 4% and 6%) of the binder material mass and in different lengths (5 mm, 10 mm and 15 mm). The addition amounts of each component of mortar are shown in Table 1. Mixing is an important part of the mortar preparation process. The purpose of mortar mixing is to achieve uniform dispersion of fibers in the mortar matrix. The mortar mixer model was the HJW-60. Before making the mortar, the fibers were soaked in water to absorb water for better bonding with the gel material. Firstly, dry fly ash, ceramic granules, expanded perlite, cement and sand were put into the mixer for 20 min. Then the wetted fiber and water were gradually put into the mixer until a homogeneous mixture was obtained. Then the mixture was poured into the mold and placed on the vibrating table for vibration and compaction, usually for about 10 s. After molding for 24 h at room temperature, it was moved into the standard maintenance box; the temperature inside the box was 20 ± 2 °C, and the relative humidity was above 95%. The moisture content, thermal conductivity and compressive strength were measured on the third, seventh and twenty-eighth days of sample conditioning. Three samples of the same mortar type were prepared for three different tests in the same period. The resistance to penetration was tested on the 28th day of sample maintenance. Six samples of the same type were prepared for conducting the resistance to penetration properties. In addition, an equal number of reference samples of ordinary mortar (without fibers) were prepared to study the effect of fibers on the mortar. The average value was taken as the result at the end of the test. Figure 2 shows the process of sample preparation.

### 2.4. Experimental Testing

**Figure 3:**
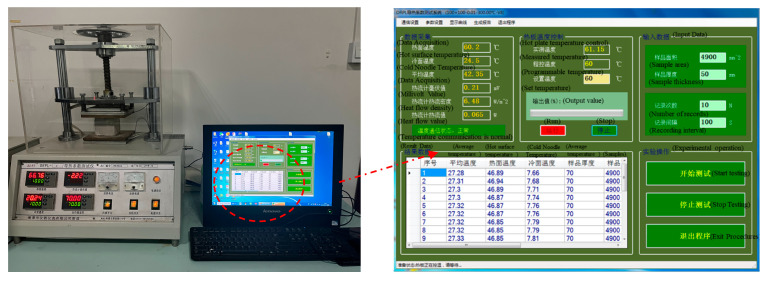
DRPL-I Thermal Conductivity Tester.

The moisture content was measured by the baking method. Different types of fiber-insulated mortars were weighed at three different periods and then immediately placed in a 105 °C oven to evaporate the water in the mortar—the mass difference between the mortar before and after heating was calculated to calculate the moisture content. Based on the steady-state plate method, the thermal conductivity of the specimens was measured using the DRPL-I thermal conductivity tester, according to, “Thermal insulation-Determination of steady-state thermal resistance and related properties-Guarded hot plate apparatus” [25]. The principle of this method is to maintain a stable temperature on the hot surface, heat transfer through the specimen to the cold surface, measure the heat flow transferred, then calculate the thermal conductivity and thermal resistance based on the thickness and heat transfer area of the specimen, and measure the thermal conductivity of the specimen with the DRPL test system. The sample is placed between the hot and cold plates of the instrument, and then the hot plate temperature and the thickness of the sample are entered into the system for testing, which takes 50–60 min for the entire test. The size of the sample used for conducting thermal conductivity tests was 100 mm × 100 mm × 50 mm. The instrument is shown in Figure 3. When straw fiber insulation mortar is used in tunnels, it is primarily a compression element, and the focus should be on its compressive strength. According to the “Standard for test method of mechanical properties on ordinary concrete,” the compressive strength of the specimens were measured using an AGX-250 electronic universal testing machine [26]. This tester has a maximum load capacity of 2000 kN and a constant rate of 1 mm/min. The dimensions of each sample used to test the compressive strength were 100 mm × 100 mm × 100 mm.

The interfacial bonding of straw fiber with concrete and the internal structure of fiber was investigated using an Apreo S HiVac Scanning electronic microscope (Semefi, Cleveland, OH, USA) with an accelerating voltage of 5 kV. The sputter coating of gold (Au) was done on samples to get quality images from SEM.

In this paper, the impermeability of fiber mortar is characterized by the method of hydrostatic pressure. The hydrostatic pressure test was conducted per the “Standard for long-term performance and durability of ordinary concrete test methods,” [27] with an SS-1.5 type concrete permeability tester. Six cone tables were made for each group, and the specific sample sizes were upper diameter 175 mm, lower diameter 185 mm and height 150 mm. Samples after 28 days of curing were tested. Starting from 0.1 MPa, the hydraulic pressure was increased by 0.1 MPa every 8 h. During the test, the surfaces of the specimens were always observed, which had been pressurized to 6 specimens in 3 specimens’ surface water seepage. Then, the maximum water pressure at this time was written, and the test was stopped. The impermeability grade is expressed by multiplying the maximum water pressure by 10. The impermeability grade is calculated according to the following Formula (1).

P = 10H − 1(1)

where P is the impermeability class and H is the water pressure when maximum water seepage occurs.

## 3. Analysis of Test Results

### 3.1. Moisture Content

**Figure 4:**
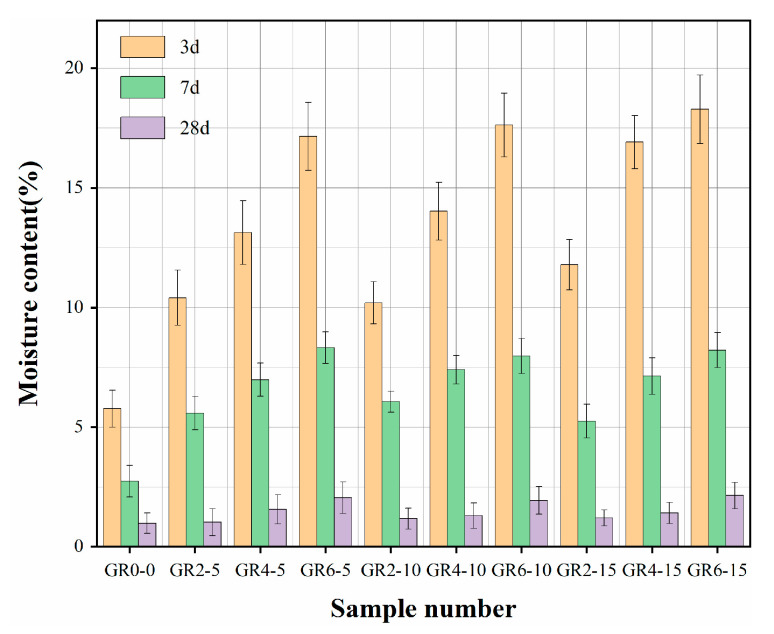
Moisture content of different types of samples. Note: GR2-5 is the abbreviation for a certain mortar sample in which 2 means that the fiber content of the mortar sample is 2%, and 5 means that the length of the fiber in the mortar sample is 5 mm. The abbreviations for other types of mortar samples have similar meaning.

The moisture contents of the ten fiber mortar samples are shown in Figure 4. The water content of each sample gradually decreased with time. After 28 days of curing, the water content increased with the increase in fiber mass. The average value ranged from 1.59% for the control sample to 3.75% for the mortar containing 6% fibers, corresponding to an increase of about 136%. This is related to the physical properties of the plant fibers, with straw fibers having a high water absorption capacity. In addition, for the mortar of the mixture to have a standard consistency, the fibers needed to absorb sufficient water before being added to the mixer. Therefore, the water content in the fibrous mortar is higher than in the control sample.

### 3.2. Thermal Conductivity

Table 2 summarizes the thermal conductivity of the fiber mortar and the reference mortar (0%). We observed that each sample’s thermal conductivity gradually decreased with time, similar to the trend of water content. On the third and seventh days, the samples had higher moisture contents, and the thermal conductivity of the fiber mortar measured at this time was generally better than that of the reference specimens. All samples had the lowest water content and the lowest thermal conductivity values on the 28th day. The mortar sample with added 10 mm and 6% fiber content had the best thermal insulation, with thermal conductivity of only 0.163 W/(m °C). Under average temperature and pressure, the thermal conductivity of liquid water is about 0.6 W/(m·°C), which has good heat transfer performance. The high water content at the early stage of mortar forming (3 d, 7 d) significantly impacts thermal insulation performance. Therefore, improving insulation materials’ water and moisture resistance is necessary. Khedari et al. have studied the hygroscopicity of plant fibers, which favors the heat transfer of composites [28].

**Figure 5:**
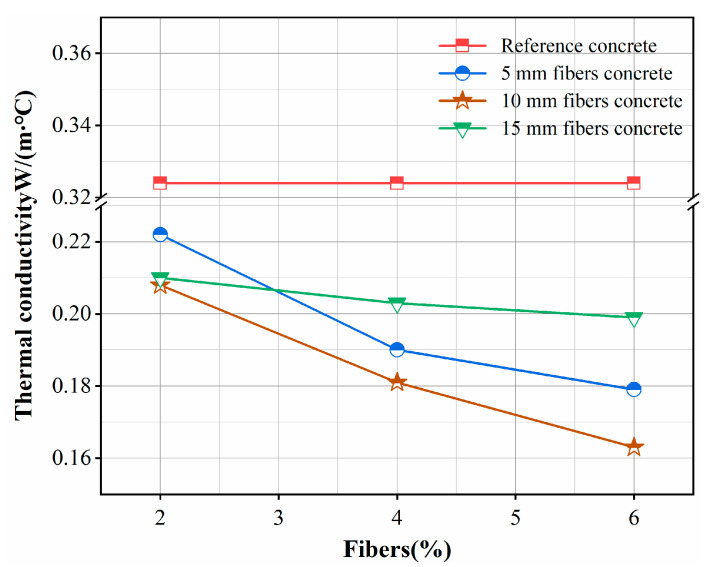
Thermal conductivity of fiber concrete (28 d).

The thermal conductivity of fiber mortar (28 d) versus fiber percentage is shown in Figure 5. This shows that the thermal conductivity of mortar with three fiber lengths decreases with the increase of fiber percentage. For the 5 mm fiber mortar, it varied from 0.222 W/(m·°C) for the GR2-5 sample, to 0.179 W/(m·°C) for the GR6-5 sample with 6% fiber content. Therefore, adding 6% fiber improved the thermal insulation performance of the mortar by approximately 44%. The same tendency was observed in 10 mm and 15 mm fiber mortars, corresponding to about 49% and 38% thermal conductivity reduction, respectively. The first caused improvement in thermal insulation due to natural ability of the plant fibers to resist heat flow because of hollow pores in their microstructure [29]. The addition of fibers also increases the number of pores in the mortar. These pores are filled with air, which is an excellent insulating material. Therefore, the higher the material’s porosity, the better the insulation performance. Foamed mortar relies on high porosity to achieve thermal insulation [30]. Secondly, rice stalk fibers have a lower thermal conductivity than cement and sand in the concrete matrix. Adding low thermal conductivity to concrete can reduce the overall thermal conductivity of the material. The aggregate type significantly affects specimens’ thermal conductivity. Omrani et al. [31] found that adding 20% Juncus acutus fiber could improve the thermal insulation performance of dry materials by about 64% and that of wet materials by about 77%.

Figure 5 also observes that the thermal conductivity of 10 mm fiber mortar was lower on day 28 for all three fiber percentage contents. The addition of 2% fibers improved the thermal insulation performance of the mortar by approximately 35%; the addition of 4% fibers improved the thermal performance of the mortar by approximately 44%; and the addition of 6% improved the thermal performance of the mortar by approximately 49%. This indicates that within a certain length, the longer the fibers are, the better the obstruction of heat flow and the lower the thermal conductivity of the mortar will be. From the point of view of thermal performance, Millogo et al. [32] have shown that the thermal conductivity of compressed earth blocks decreases with increasing content and length of hibiscus cannabinus fibers. The thermal insulation performance of 15 mm fiber mortar is relatively poor. By observing the distribution of fibers in the specimen’s cross-section, 15 mm fibers are bent and folded, which can easily form too-large pores. Too-long flat fibers formed by the tiny pores will be connected into channels. Convective heat transfer of air within larger pores and channels will be enhanced, as will radiative heat transfer between walls, which will increase the thermal conductivity of the mortar [33]. Therefore, the addition of too-long fibers in the mortar will, conversely, weaken the insulation performance.

### 3.3. SEM Micrographs

**Figure 6:**
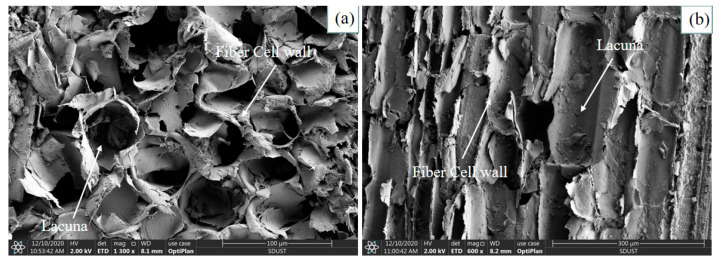
SEM micrographs of (**a**) cross-section of Rice straw fiber, (**b**) longitudinal section of straw fiber epidermis.

Figure 6 reports representative scanning electron microscopy (SEM) images of single rice straw fiber. Both cross-sectional images and longitudinal-sectional images are shown. The rice straw fiber tissue shows a highly porous network with an overall honeycomb shape in Figure 6a. The internal cell wall structure of the fibers is dense, and the lacuna is small, almost circular, with a diameter of about 30–50 μm. The epidermis (Figure 6b) of the fiber consists of many rectangular lacunas, neatly arranged, with the same length direction as the fiber axially, and the length is about 150–300 μm. Most straw fibers show a similar structure with a lacuna in the center, surrounded by cell walls [34]. The interior of the straw fiber tissue shows a high porosity, which explains its good thermal insulation performance. These findings are similar to the observations of Jorge Pinto et al. [35].

**Figure 7:**
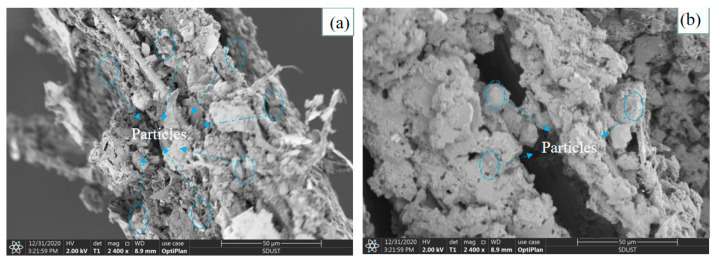
Fiber–matrix binding interface (left) and fiber surface (right) of (**a**) SEM micrographs of the mortar with 2% fibers and (**b**) SEM micrographs of the mortar with 6% fibers.

The chemical composition of straw fibers contains wax, which is concentrated in the waxy layer on the outer surface of the fiber. The waxy layer has various functions, such as retaining moisture under continuous light on sunny days, protecting the plant from sun damage and protecting the plant from leaf-eating pests. However, the native surface of the fiber is smooth due to the presence of the waxy layer. Therefore, in fiber manufacturing engineering, we treat the fiber with NaOH solution to increase the roughness of the fiber surface. Figure 7 shows the SEM images collected from concretes. Figure 7a shows the SEM images of the fiber–matrix binding interface and the fiber surface collected from mortar samples with low fiber content (GR2-5). In terms of bonding, the interfacial region of the fiber–matrix interface shows that the bonding appears to be high, with the fibers tightly bonded to the matrix and partially embedded in the matrix. A good cement surface coverage was observed on the fiber surface, and cement and fly ash particles uniformly adhered to the fiber surface. This phenomenon is due to the high roughness of the fiber surface, which is expected to improve the mechanical interlocking with the mortar matrix [34]. In addition, the high porosity of the rice stalk fiber surface (Figure 7a) promotes the penetration of the gel material and, thus, the formation of anchor points.

Figure 7b shows SEM images of the fiber–matrix bonding interface and fiber surface collected from mortar samples with high fiber content (GR6-15). There are voids at the fiber–matrix interface, and the degree of bonding appears to be relatively low. Small amounts of cement and fly ash particles adhered unevenly to the fiber surface. It was found that if the fiber content was too large or the length was relatively long, the fibers would intertwine with each other, and the fibers were not sufficiently mixed with other raw materials. This phenomenon resulted in the gel material not sufficiently wrapping the fibers.

### 3.4. Compressive Strength

**Figure 8:**
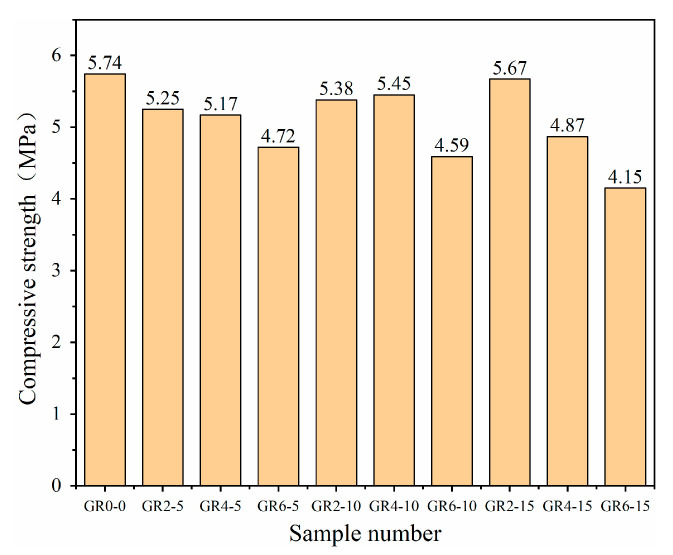
Compressive strength of various types of mortar on the third day.

**Figure 9:**
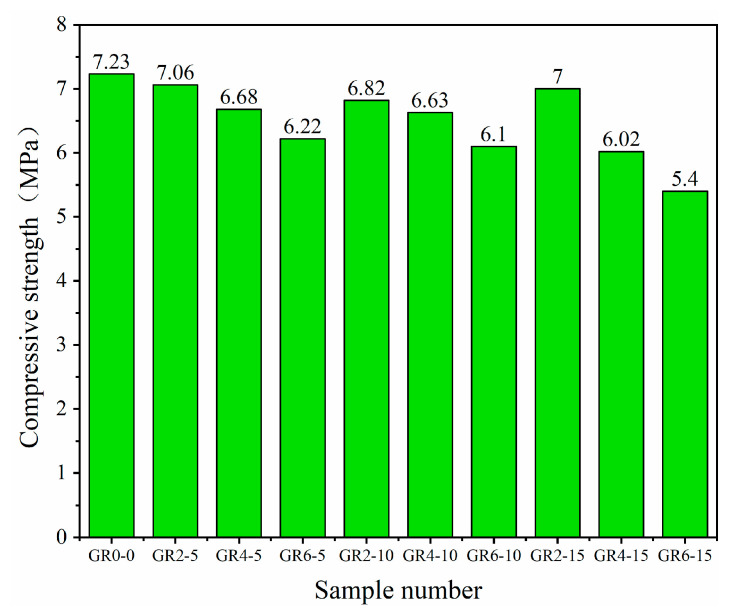
Compressive strength of various types of mortar on the seventh day.

**Figure 10:**
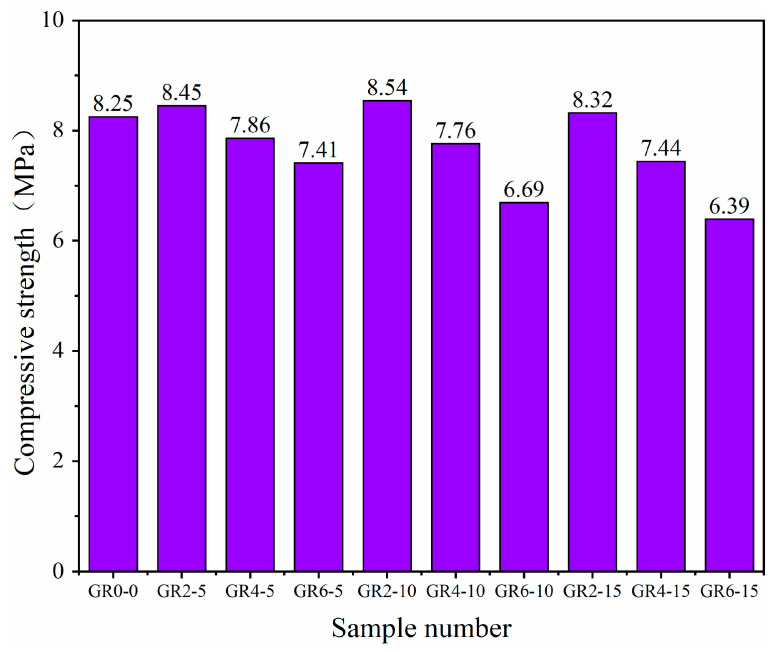
Compressive strength of various types of mortar on the twenty-eighth day.

**Figure 11:**
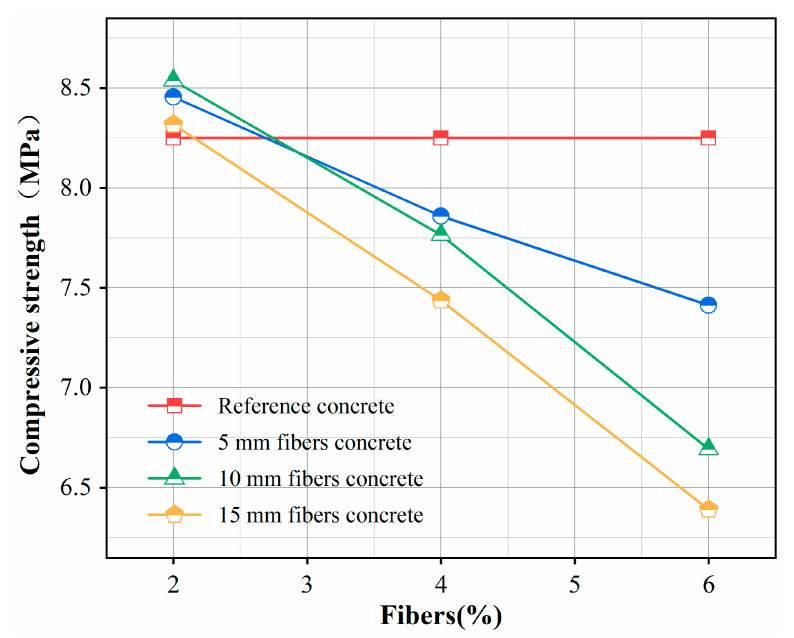
Compressive strength of the fiber insulation mortar measured at day 28.

Figure 8, Figure 9 and Figure 10 show the compressive strength of the 10 types of mortar samples tested on the third, seventh and twenty-eighth days, respectively. Comparing the three ages of mortar, we observed that the added rice stalk fibers reduced the early strength of the mortar. In this case, the compressive strength of the mortar with the addition of 10 mm, 2% fiber was reduced by 3.21% and 5.37% on the third and seventh days, respectively. The compressive strength of mortar with three different lengths (5 mm, 10 mm and 15 mm) of fibers added on day 28 as a function of fiber percentage is shown in Figure 11. It can be observed that mortar with three lengths of fibers shows the same trend; compressive strength decreases with increasing fiber content. For example, the addition of fibers reduced the compressive strength of the mortar from 8.25 MPa to 7.413 MPa when the content of 5 mm fibers was increased from 0% to 6%. However, the decrease in compressive strength was not proportional to the fiber content, and there was no significant correlation with the fiber length.

The compressive strength of the mortar was improved when 2% fiber was added. The compressive strength of mortar with 5 mm, 10 mm and 15 mm fibers increased by 2.48%, 3.49% and 0.82%, respectively. A small amount of fiber acts as reinforcement in fiber mortar. Straw fibers have lower compressive strength than sand and ceramsite but can hinder the lateral strain during the compression, increasing compressive strength. The presence of more fibers may have some negative impact on the strength. The compressive strengths of the mortar with 4% and 6% fibers were all lower than the strengths of the reference samples. The lowest compressive strength (6.39 MPa) of mortar with 6% and 15 mm fibers added showed a 22.5% strength decline. When there is an excess of fibers, the distribution is not uniform, and the fibers overlap due to the bundling effect—this phenomenon results in little gel material between the fibers, which introduces voids and discontinuities. When the mortar is loaded, cracks develop around the fibers, accelerating the destruction of the matrix. In addition, the easy bending of 15 mm length fibers and the non-uniform alignment of fibers with the transverse tension direction are also factors of strength reduction. As observed by other researchers, the above compressive strength mechanisms are typical of natural-fiber-reinforced composites [36,37].

### 3.5. Impermeability Grade

**Figure 12:**
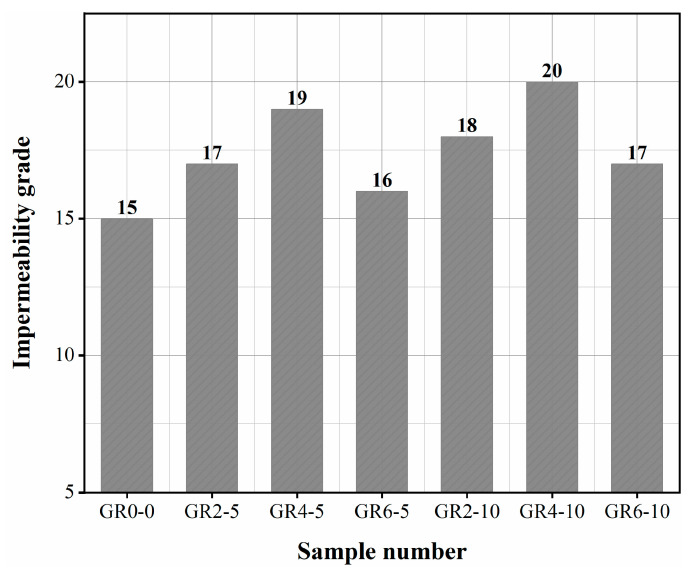
Impermeability grade of mortar.

After compressive strength tests and thermal conductivity tests, it was found that the thermal insulation properties and compressive strength of mortar mixed with 15 mm length fibers were severely reduced. Therefore, the impermeability test mainly considered the effect of 5 mm and 10 mm length fibers on the impermeability performance. The impermeability grade of fiber mortar and reference sample (GR0-0) is shown in Figure 12. Figure 12 shows that fiber concrete’s impermeability was significantly higher than that of the reference mortar. GR4-10’s impermeability level reached 20, 1.3 times GR0-0’s. Glass beads are a porous material with low strength and high permeability. Expanded perlite in mortar matrix tended to form seepage channels under higher water pressure and enhanced the permeability of mortar. Stalk fiber better compensates for the disadvantages of expanded perlite. The impermeability of fiber mortar does not increase with the increase of fiber content. When the fiber content was 4%, the impermeability was the best. Too many fibers tend to tangle and overlap, forming excessive voids that provide channels for water infiltration, resulting in poor impermeability; similar findings were found in the thermal conductivity and compressive strength tests.

### 3.6. Comprehensive Evaluation

Adding 2% content and 10 mm fiber to the mortar improved thermal insulation and compressive strength with good impermeability. After 28 days of maintenance, the thermal conductivity of 2% content, 10 mm fiber insulation mortar was 0.208 W/(m °C), the compressive strength was improved by 3.49% and the impermeability grade was 18, with the most excellent overall performance. GR2-10 was the best ratio of fiber insulation mortar mix.

## 4. Simulation of the Thermal Insulation

Currently, both strong tunnels and broken high-temperature tunnels are sprayed with normal concrete and then mechanically ventilated to cool them down. Certain tunnels with stable surrounding rocks and which are very solid should be supported by spraying fiber insulation mortar with excellent insulation properties. This type of tunnel is so strong that it does not need ordinary concrete with high compressive strength, but it needs insulation mortar to cool it down. Using a numerical simulation, we aimed to verify the necessity of spraying thermal insulating mortar in solid tunnels by comparing the cooling effect of spraying plain concrete and stalk fiber thermal insulating mortar (GR2-10) on the tunnel surface.

### 4.1. Numerical Simulation Methodology

#### 4.1.1. Building Geometric Models

**Figure 13:**
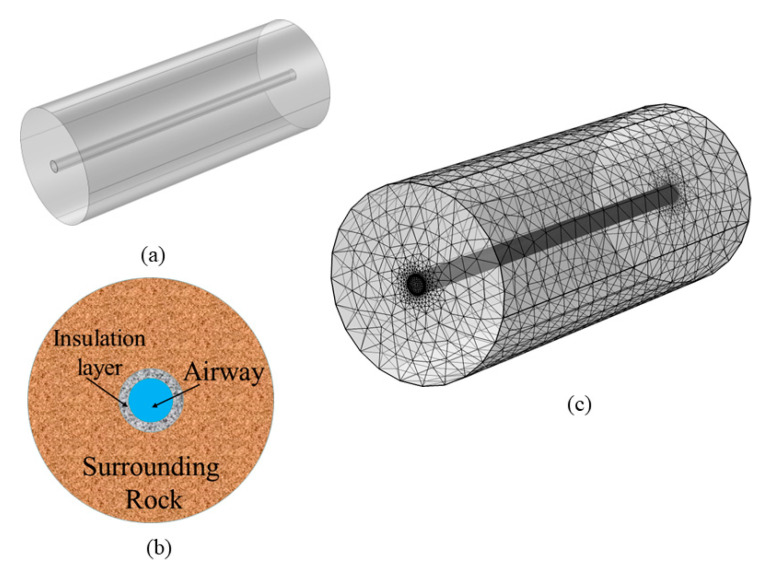
Geometric model: (**a**) Three-dimensional stereo view. (**b**) Cross-section. (**c**) Meshing.

This study is based on a section of a concentrated flat tunnel located at a gold mine in Shandong Province, China, at a depth of 1020 m underground. The geometry of the numerical model is a semi-infinite three-dimensional hollow cylinder of similar size to the actual alleyway (Figure 13a). The cross-section of the model consists of the airflow, insulation and the surrounding rock (Figure 13b). The length of the model is 100 m; the sizes of the airflow, insulation and surrounding rock are 2 m, 0.2 m and 17.8 m, respectively. The mesh division of the 3D model is shown in Figure 13c; the mesh volume is 1.246 × 10^−5^ m^3^, where the mesh cells consist of tetrahedra, prisms, quadrilaterals and triangles, and the total number of mesh cells is 5.89905 × 10^5^. The minimum cell mass, average cell mass and cell volume ratio are 0.1394, 0.6584 and 2.005 × 10^−5^, respectively.

#### 4.1.2. Fundamental Assumptions

The main assumptions, which are commonly applied to the rock–airflow conjugated heat transfer model, were as follows [38]:

1. The thermal physical properties of the surrounding rock and insulation layer are assumed stable, homogeneous and isotropic;
2. The incompressible ideal gas law was applied to define the fluid density;
3. The heat loss caused by the work done by the viscous force of the fluid was ignored;
4. The airflow was fully developed and turbulent;
5. The turbulent viscosity was isotropic, and the turbulent viscosity coefficient could be considered as a scalar;
6. The thermal continuity involving stationary and no-slip conditions was applied to the outer surface of the heading and between the inner surface of the airway and the air;
7. The constant temperature, which could be regarded as the virgin rock temperature, was applied to the far-field boundary condition.

#### 4.1.3. Governing Equations

The conjugate heat transfer system between the surrounding rock and the airflow is divided into two parts: heat conduction and convection. Heat conduction occurs in the rock and insulation around the tunnel, while heat convection occurs mainly within the airflow and at the boundary between the airflow and the tunnel wall. Their related equations are as follows.

Heat transfer equation

(2) $$\rho C_{P}\frac{\partial T}{\partial t}+\nabla \cdot q=Q$$

where ρ is the density of the insulation layer and surrounding rock; $C_{P}$ is the specific heat capacity of the insulation layer and surrounding rock; *T* is the temperature of the insulation layer and surrounding rock; *t* is time; *q* is the heat flux density of the insulation layer and surrounding rock; Q is the heat source of the insulation layer and surrounding rock; and λ is the thermal conductivity of the insulation layer and surrounding rock.

Heat convection equation

Among the commonly used turbulence models, the tunnel airflow temperature calculated by the *k*-*ε* model is closest to the actual situation. It provides better results for the simulation of airflow temperature. In the tunnel flow, turbulent mass, momentum and energy transport coincide. The mass, momentum and energy conservation equations can be expressed as follows:

Continuity equation

(3) $$\frac{\partial \rho }{\partial t}+\nabla \cdot \left(\rho U\right)=0$$

Momentum equation

(4) $$\frac{\partial }{\partial t}\left(\rho U\right)+\nabla \cdot \left(\rho UU\right)=-\nabla P+\nabla \cdot (\overset{=}{\tau })+\rho \vec{g}$$

where ρ is air density, *P* is the static pressure, U is the velocity vector, $\overset{=}{\tau }$ is the stress tensor and $\rho \vec{g}$ is the gravitational body force.

Energy conservation equation

(5) $$\frac{\partial }{\partial t}\left(\rho E\right)+\nabla \cdot \left(U\left(\rho E+\rho \right)\right)=-\nabla \cdot \left(\sum_{j}h_{j}J_{j}\right)+S_{q}$$

where *E* is total energy, $h_{j}$ is the formation enthalpy of species *j*, $J_{j}$ is the diffusion flux of species *j* and $S_{q}$ includes the heat of chemical reaction, and any other volumetric heat sources.

*k*-*ε* two equation turbulence model

(6) $$\frac{\partial }{\partial t}\left(\rho k\right)+\nabla \cdot \left(\rho Uk\right)=\nabla \cdot \left[\left(\mu +\frac{\mu_{t}}{\sigma_{k}}\right)\nabla k\right]+G_{k}-\rho \varepsilon$$

(7) $$\frac{\partial }{\partial t}\left(\rho \varepsilon \right)+\nabla \cdot \left(\rho U\varepsilon \right)=\nabla \cdot \left[\left(\mu +\frac{\mu_{t}}{\sigma_{\varepsilon }}\right)\nabla \varepsilon \right]+C_{1\varepsilon }\frac{\varepsilon G_{k}}{k}-C_{2\varepsilon }\rho \frac{\varepsilon^{2}}{k}$$

(8) $$\mu_{t}=\rho C_{\mu }\frac{k^{2}}{\varepsilon }$$

where $G_{k}$ represents the generation of turbulence kinetic energy due to the mean velocity gradients, *μ* the dynamic viscosity of the fluid, $C_{1\varepsilon }$ and $C_{2\varepsilon }$ are model constants, $\sigma_{k}$ and $\sigma_{\varepsilon }$ are the turbulent Prandtl numbers corresponding to the *k* equation and the *ε* equation, respectively, and $\mu_{t}$ is turbulent viscosity.

#### 4.1.4. Boundary Conditions and Parameters

The boundary conditions were set according to the actual situation on site. The heat source in the tunnel is mainly the rock around the tunnel, and the primary type of rock is siltstone. The temperature of the outermost boundary was set as the initial temperature of the rock (50 °C). The gas flow velocity at the inlet was 2.5 m/s, and the temperature was 15.5 °C. Fluid flow and solid heat transfer are coupled by conjugate heat transfer. The tunnel’s walls greatly influence the turbulent flow state and, thus, the heat exchange. In this study, the effect of roughness was considered by correcting the wall roughness by specifying two roughness parameters: the roughness height *Ks* and the roughness constant *Cs*. The settings of the boundary condition parameters in the simulation are shown in Table 3. The thermophysical parameters of the surrounding rock, airflow, plain concrete and fiber insulation mortar are shown in Table 4.

### 4.2. Thermal Insulation Effect

**Figure 14:**
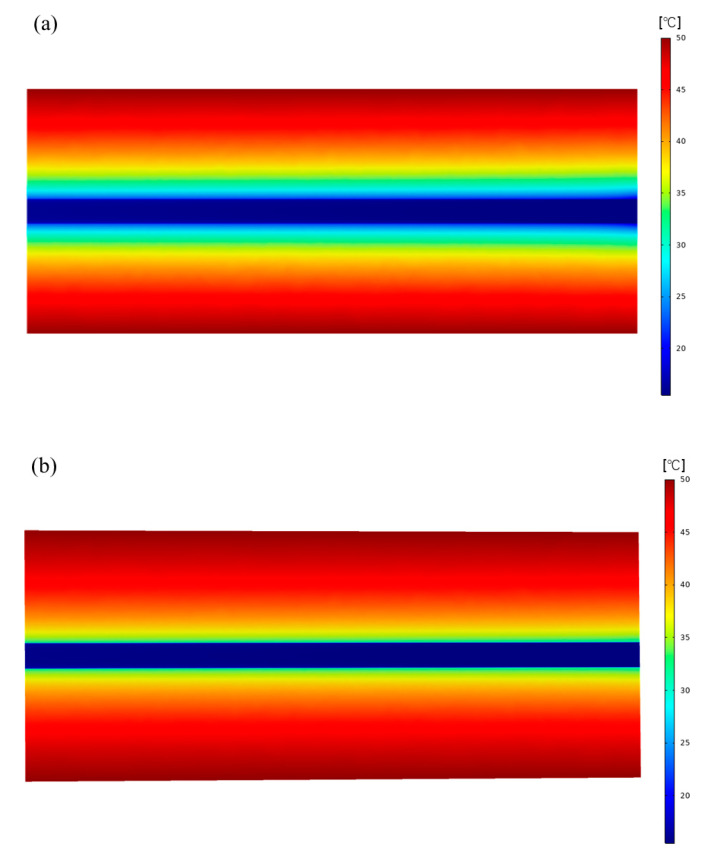
Longitudinal section cloud diagram of temperature field of surrounding rock: (**a**) Temperature field cloud of plain mortar supported tunnel. (**b**) Temperature field cloud of insulated mortar supported tunnel.

The temperature distribution on the longitudinal section of the surrounding rock was studied, as shown in Figure 14. As seen in Figure 14a, the rock temperature in the vicinity of the tunnel wall drops significantly due to the fresh air flow cooling effect. This cooling effect diminishes as it moves further away from the roadway. When the distance exceeds a certain threshold, the rock temperature remains almost at the original rock temperature (50 °C), which indicates that the range of airflow perturbation of the surrounding rock temperature field is limited, and the heat flux is stable after a period of ventilation. During the airflow, there is a significant drop in rock temperature near the entrance. After a certain distance, the decrease in rock temperature gradually decreases. This is because the temperature difference between the airflow and the surrounding rock at the entrance is the largest, the heat exchange is intense and the temperature difference gradually decreases with the increase of the ventilation distance. Figure 14b shows the temperature field distribution of the surrounding rock in the tunnel supported by insulated mortar. The surrounding rock’s temperature field is less disturbed, and the overall surrounding rock temperature is higher than the tunnel supported by plain concrete. Due to the small thermal conductivity, the insulated mortar keeps the heat out of the airflow and reduces the heat transfer from the surrounding rock to the airflow.

**Figure 15:**
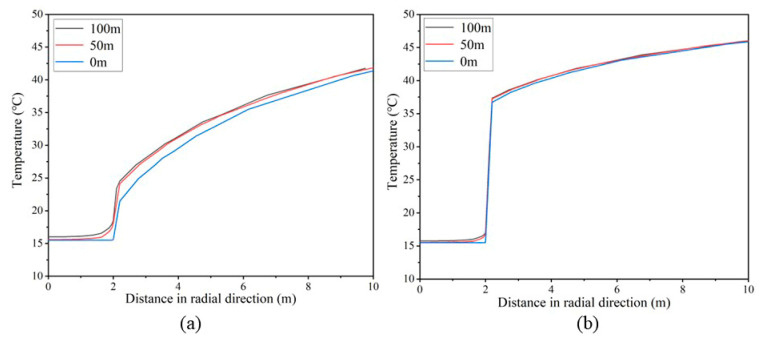
Temperature distribution along the central radial direction: (**a**) Tunnels supported by plain mortar. (**b**) Tunnels supported by insulated mortar.

Figure 15 shows the temperature distribution curves in the center radial direction at the entrance (0 m), middle (50 m) and exit (100 m) of the tunnel. As can be seen in Figure 15, the temperatures at the tunnel inlet and outlet are the lowest and highest, respectively. From the center of the tunnel to the interior of the surrounding rock, the temperature rises very slowly in the airflow, accelerates in the concrete, and rises steadily in the surrounding rock. Since the thermal conductivity of concrete is between air and surrounding rock, the concrete layer is the transition layer for the whole heat flow, and the temperature varies tremendously in the concrete layer. The temperature difference between plain concrete is about 8 °C, the temperature difference between insulated concrete is about 20 °C and the internal temperature difference between the two types of concrete is 12 °C. Insulated mortar can effectively block the heat transfer between the airflow and the surrounding rock.

**Figure 16:**
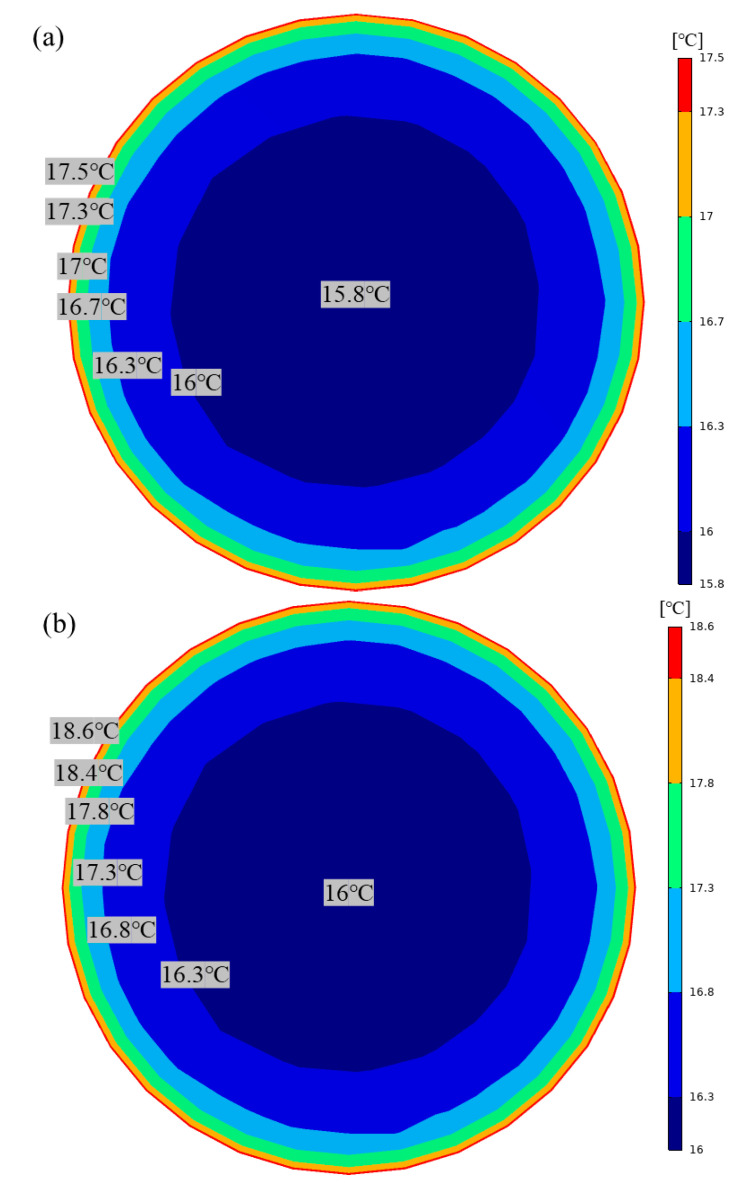
Temperature field of air flow at the exit of the roadway: (**a**) Temperature field cloud of plain mortar supported tunnel. (**b**) Temperature field cloud of insulated mortar supported tunnel.

Figure 16 shows the temperature field distribution on the cross-section of the airflow exit after a ventilation distance of 100 m. The temperature field is expressed as two regions along the radial direction: the central and growth regions. As shown in Figure 16a, the airflow temperature in the central region is kept at the lowest state, with a slow temperature growth and a temperature gradient of only 0.5 °C/m. The airflow temperature near the tunnel wall is higher than the airflow temperature near the center, and the temperature proliferates with a temperature gradient of 6.3 °C/m, where the closer to the wall, the faster the temperature growth rate. This is because the heat transfer in the growth area near the wall is convective heat transfer consisting of heat conduction and thermal convection. The temperature difference between the roadway’s wall and the airflow is significant, which is conducive to heat transfer. On the other hand, the wall surface is rough, and when the airflow passes through, it creates a smaller vortex, which facilitates thermal convection. The direction of airflow in the central area of the roadway is perpendicular to the direction of heat transfer in the radial direction, which is very unfavorable for heat convection, resulting in a smaller temperature gradient in the central area. Figure 16b shows the airflow temperature distribution at the tunnel’s exit supported by insulated mortar. Due to the presence of insulating mortar, which slows down the heat transfer from the surrounding rock to the airflow, the temperature gradient is smaller than that of a tunnel supported by plain concrete, with 0.3 °C/m and 5.2 °C/m in the central and growth zones, respectively. The average temperature of the exit airflow of the insulated mortar supported tunnel is 16.2 °C, which is 0.5 °C lower than the average temperature of the exit airflow of plain concrete. Therefore, excellent insulation performance can be obtained when insulating mortar is sprayed on the tunnel walls.

## 5. Conclusions

Performance tests and numerical simulations analyzed straw fiber mortar’s physical properties and thermal insulation effects for tunnel insulation. The following conclusions can be drawn.

1. According to the results, the moisture content of the samples was relatively high during the initial stages of mortar formation. The evaluation of the thermal insulation properties highlighted the detrimental effect of a high moisture content in the mortar, in that the high water content weakened the thermal insulation properties. On day 28, the thermal conductivity of the mortar mixed with three different lengths of fibers decreased with an increasing percentage of fibers. The thermal insulation performance of the 5 mm, 10 mm and 15 mm mortars increased by about 44%, 49% and 38%, respectively, when the fiber admixture was 6%. The fiber mortar with a 10 mm length has the best thermal insulation performance among mortars of the same length type, and the thermal conductivity of mortar with 6% content is as low as 0.163 W/(m °C); the longer the fiber is in a certain length range, the better the effect on the obstruction of heat flow.
2. The SEM micrographs show that the mortar with 2% content has a uniform distribution of gel material on the fiber surface, and the fibers are tightly bonded to the matrix, making the 5 mm, 10 mm and 15 mm fibers of the mortar compressive strength increased by 2.48%, 3.49% and 0.82%, respectively. With increased fiber content (4%, 6%), voids and discontinuities were generated due to the binding effect, resulting in a decrease in compressive strength. The mortar with 6% added 15 mm fibers had the lowest compressive strength (6.39 MPa), showing a 22.5% decrease in strength. In addition, adding fibers can effectively improve the impermeability of the mortar.
3. By comparing the simulation results for the cooling effect of straw fiber insulation mortar with the GR2-10 proportion and ordinary concrete, it can be seen that the difference in internal temperature between the two types of materials is 12 °C, while the difference in temperature at the outlet is 0.5 °C. The straw fiber insulation mortar sprayed on the surface of the high-temperature tunnel can exert an effective heat insulation effect, which effectively hinders the transfer of heat flow to the airflow.

## Figures and Tables

**Table 1:** Density of mortar components.

Materials	The Addition Amounts of Each Component of Mortar (kg/m^3^)									
	GR0-0	GR2-5	GR4-5	GR6-5	GR2-10	GR4-10	GR6-10	GR2-15	GR4-15	GR6-15
Cement	26.24	26.24	26.24	26.24	26.24	26.24	26.24	26.24	26.24	26.24
Fly ash	6.56	6.56	6.56	6.56	6.56	6.56	6.56	6.56	6.56	6.56
Sand	75.44	75.44	75.44	75.44	75.44	75.44	75.44	75.44	75.44	75.44
Ceramic granules	42.65	42.65	42.65	42.65	42.65	42.65	42.65	42.65	42.65	42.65
Glass beads	29.15	29.15	29.15	29.15	29.15	29.15	29.15	29.15	29.15	29.15
Water	18.04	18.04	18.04	18.04	18.04	18.04	18.04	18.04	18.04	18.04
Fibers	0	0.65	1.32	1.97	0.65	1.32	1.97	0.65	1.32	1.97

**Table 2:** Thermal conductivity of Reference specimen mortar and fibers mortar.

Time	Thermal Conductivity (W/(m °C))									
	GR0-0	GR2-5	GR4-5	GR6-5	GR2-10	GR4-10	GR6-10	GR2-15	GR4-15	GR6-15
3d	0.647	0.696	0.934	1.178	0.674	0.911	1.004	0.710	1.076	1.138
7d	0.535	0.615	0.873	0.865	0.561	0.846	0.811	0.500	0.779	0.815
28d	0.324	0.222	0.190	0.179	0.208	0.181	0.163	0.210	0.203	0.199

**Table 3:** Setting of simulation parameters.

Type	Property	Value	Type	Property	Value
Inlet	Velocity inlet	2.5 m/s	Wall of airway	*Ks*	0.05 m
	Temperature	15.5 °C		*Cs*	0.07
Outlet	Pressure outlet	1.1 atm	Solution methods	Scheme	Coupled
Viscous model	*K*-epsilon	Standard	Spatial discretization	Turbulent kinetic energy	Second-order upwind
Far-Field boundary	Temperature	\		Turbulent dissipation rate	Second-order upwind
General	Solver type	Pressure-based		Pressure	PRESTO!

**Table 4:** Thermophysical parameters of the surrounding rock, the support and the air.

Material	Thermal Conductivity (W/(m∙°C))	Density (kg/m^3^)	Specific Heat (J/(kg °C))
surrounding rock	5.1	2593	790
plain concrete	1.5	2200	900
insulating mortar	0.208	1650	\
air	0.0242	1.225	1006.43

## Data Availability

The study did not report any data.
